# Discovering the Influence of Microorganisms on Wine Color

**DOI:** 10.3389/fmicb.2021.790935

**Published:** 2021-12-03

**Authors:** Rosanna Tofalo, Giovanna Suzzi, Giorgia Perpetuini

**Affiliations:** Faculty of Bioscience and Technology for Food, Agriculture and Environment, University of Teramo, Teramo, Italy

**Keywords:** wine, color, yeasts, lactic acid bacteria, fermentation, metabolism, polyphenols

## Abstract

Flavor, composition and quality of wine are influenced by microorganisms present on the grapevine surface which are transferred to the must during vinification. The microbiota is highly variable with a prevalence of non-*Saccharomyces* yeasts, whereas *Saccharomyces cerevisiae* is present at low number. For wine production an essential step is the fermentation carried out by different starter cultures of *S. cerevisiae* alone or in mixed fermentation with non-*Saccharomyces* species that produce wines with significant differences in chemical composition. During vinification wine color can be influenced by yeasts interacting with anthocyanin. Yeasts can influence wine phenolic composition in different manners: direct interactions—cell wall adsorption or enzyme activities—and/or indirectly—production of primary and secondary metabolites and fermentation products. Some of these characteristics are heritable trait in yeast and/or can be strain dependent. For this reason, the stability, aroma, and color of wines depend on strain/strains used during must fermentation. *Saccharomyces cerevisiae* or non-*Saccharomyces* can produce metabolites reacting with anthocyanins and favor the formation of vitisin A and B type pyranoanthocyanins, contributing to color stability. In addition, yeasts affect the intensity and tonality of wine color by the action of β-glycosidase on anthocyanins or anthocyanidase enzymes or by the pigments adsorption on the yeast cell wall. These activities are strain dependent and are characterized by a great inter-species variability. Therefore, they should be considered a target for yeast strain selection and considered during the development of tailored mixed fermentations to improve wine production. In addition, some lactic acid bacteria seem to influence the color of red wines affecting anthocyanins’ profile. In fact, the increase of the pH or the ability to degrade pyruvic acid and acetaldehyde, as well as anthocyanin adsorption by bacterial cells are responsible for color loss during malolactic fermentation. Lactic acid bacteria show different adsorption capacity probably because of the variable composition of the cell walls. The aim of this review is to offer a critical overview of the roles played by wine microorganisms in the definition of intensity and tonality of wines’ color.

## Introduction

Wine market is facing several challenges due to consumer demands for high quality wines. The quality of a wine depends on several factors, including grape variety, soil management, winemaking techniques, alcoholic strength, residual sugar content, total and volatile acidity, aroma, flavor, astringency, bitterness, and color. In fact, color intensity and tonality are considered one of the main parameters contributing to the quality of wine and a matter of concern to winemakers (Forino et al., 2020). In general, the color of young red wines mainly relies on the concentration of monomeric anthocyanins and related compounds, which are extracted from grape skins during the maceration process (Ribereau-Gayon et al., 2006). Polymeric pigments and anthocyanin-derived compounds such as visitins are more resistant to bisulfite bleaching and oxidation and are the main responsible of observed color in aged red wines (Morata et al., 2016). However, a reduction of their concentration occurs during aging and storage of red wine, because of the conversion of monomeric anthocyanins to polymeric pigments and the formation of anthocyanin derivatives (Morata et al., 2016). Wine color is influenced by several factors including the grapevine variety, agricultural practices, and fruit maturation, as well as oenological protocols, such as destemming and crushing conditions, yeast strains used for alcoholic fermentation, malolactic fermentation (MLF), maceration procedures, and wine aging (Ribereau-Gayon et al., 2006; Morata et al., 2016). Yeasts play a key role in the definition of wine color. In fact, they can reduce color intensity and modify wine tonality by deglycosylation of anthocyanins catalyzed by β-glycosidase or anthocyanidase enzymes (Manzanares et al., 2000), through the direct adsorption of pigments on yeasts’ cell wall, and producing metabolites such as pyruvic acid and acetaldehyde that have been found to react with different phenolic compounds (Morata et al., 2003, 2006, 2016; Medina et al., 2005; Caridi et al., 2007). Yeast adsorption and its impact on wine color has been demonstrated in several studies and actually is considered an important target for yeast selection. Wine color can also be affected by the metabolic activity of lactic acid bacteria (LAB) (Devi and Ka, 2019). In fact, color loss is common in wines that have undergone MLF (Virdis et al., 2021). LAB can also liberate hydroxycinnamic acids from their tartaric esters and have the potential to break down anthocyanin glucosides, thus impacting wine color (Virdis et al., 2021). This review focuses on the role of wine microorganisms in the definition of wine color.

## Wine Fermentation

Wine fermentations are characterized by a heterogeneous microbiota and yeasts play a major role in this process. This complex microbial array influences the characteristics of the final product thanks to the coexistence and succession of different species/strains along the fermentation process. From a microbiological point of view, winemaking involves two main steps, the alcoholic fermentation (AF) and MLF. Alcoholic fermentation, mainly driven by *Saccharomyces cerevisiae*, leads to the formation of metabolites of oenological interest (Álvarez-Pérez et al., 2012). However, recent studies demonstrated that hybrids with other species of the *Saccharomyces* complex (e.g., *S. bayanus*, *S. kudriavzevii*, and *S. mikatae*) showed similar fermentation power and vigor and sometimes are preferred in fermentation trials (Gonzalez et al., 2006; Bellon et al., 2013; Peter et al., 2018). However, despite the predominant status of *S. cerevisiae*, many non-*Saccharomyces* (NS) yeasts participate to wine fermentation and can shape the sensory characteristics of the wines. In fact, these yeasts may influence the production of secondary and volatile compounds such as esters, higher alcohols, acids and monoterpenes increasing wine quality and complexity (for a review see Padilla et al., 2016). Their occurrence in wine environment has been known for more than a 100 years but they have been considered as spoilage microorganisms or irrelevant species. Thanks to the microbiological studies performed during the last decades enriched with the help of metataxonomic studies (Setati et al., 2012; Bokulich et al., 2013) their role in winemaking has been reconsidered. According to Jolly et al. (2014) NS yeasts can be divided into 3 groups: (i) aerobic yeasts such as *Candida* spp., *Cryptococcus* spp., *Debaryomyces* spp., *Pichia* spp., and *Rhodoturula* spp.; (ii) low fermentative yeasts including *Hanseniaspora uvarum* (*Kloeckera apiculata*), *Hanseniaspora guilliermondii* (*Kloeckera apis*), and *Hanseniaspora occidentalis* (*Kloeckera javanica*); (iii) fermentative yeasts e.g., *Kluyveromyces marxianus* (*Candida kefyr*), *Metschnikowia pulcherrima* (*Candida pulcherrima*), *Torulaspora delbrueckii* (*Candida colliculosa*), and *Zygosaccharomyces bailii*.

*S. cerevisiae* and NS yeast species do not simply passively coexist during wine fermentation, but a metabolic interplay occurs between them. For instance, mixed fermentations between *S. cerevisiae* and *T. delbrueckii* and *H. vineae*, seem to be a good strategy to enhance wine aroma diversity (Liu et al., 2019). Moreover, *Starm. bacillaris* (syn. *C. zemplinina*) if used in mixed fermentation with *S*. *cerevisiae*, improve the fermentation kinetic with low ethyl acetate and acetic acid production (Tofalo et al., 2016). Therefore, it is essential not only to select yeasts with suitable oenological properties, but also to consider other aspects including inoculation density, timing, and combination of strains in the organoleptic properties of wines (Englezos et al., 2018). Several efforts must be undertaken in order to establish a link between an inoculation protocol and the chemical composition as well as the chromatic characteristics of wines using the same couple of strains and fermentation conditions. Table 1 reports the main activities of non-*Saccharomyces* yeasts during wine fermentation and the inoculation protocols applied.

**TABLE 1:** Main roles of non-*Saccharomyces* yeasts in winemaking.

Species	Inoculation strategy	Role in winemaking	References
*T. delbrueckii*/ *S. cerevisiae*	Sequential	High production of terpenols, and 2-phenylethanol, higher concentrations of thiols	Bely et al., 2008; Renault et al., 2009, 2016; Comitini et al., 2011; Sadoudi et al., 2012; Van Breda et al., 2013; Belda et al., 2017; Benito, 2018
	Co-inoculation	Low volatile acidity	
*Starm. bacillaris/S. cerevisiae*	Co-inoculation	Reduced amount of acetic acid	Tofalo et al., 2012; Lencioni et al., 2018
	Co-inoculation, sequential	High production of glycerol production and low ethanol yield, acetic acid decrease	
*L. thermotolerans/S. cerevisiae*	Co-inoculation, sequential	Production of lactic acid, 2-phenylethanol, glycerol, and polysaccharides	Gobbi et al., 2013; Benito et al., 2016; Vilela, 2018
*M. pulcherrima/S. cerevisiae*	Sequential	Production of volatile terpene and varietal thiols	Barbosa et al., 2018
	Co-inoculation	Acetic acid decrease	
	Co-inoculation, sequential	Ethyl ester increase	
*Sch. pombe/S. cerevisiae*	Co-inoculation, sequential	Reduction of malic acid amount, production of pyruvic acid and polysaccharides	Benito et al., 2012, 2014; Domizio et al., 2017, 2018

*Modified from Padilla et al. (2016).*

Lactic acid bacteria are responsible of MLF which usually takes place after the AF. Malolactic fermentation is a process required for most red wines and some white wines; it consists of decarboxylation of the L-malic acid to L-lactic acid and induces pH increase, makes wines more palatable by reducing the sour taste associated to malic acid, and provides additional advantages, like microbial stability and improved aroma complexity (Virdis et al., 2021). In particular, LAB belonging to *Lactiplantibacillus, Pediococcus, Leuconostoc*, and *Oenococcus* genera drive the MLF. They are also involved in the definition of wine aroma releasing diacetyl, esters and volatile thiols. They also show pectinolytic activity, which could be useful to improve clarification and the ability to break down acetaldehyde (Virdis et al., 2021). Moreover, recent studies highlighted their role in the definition of wine color (Virdis et al., 2021).

Several studies highlighted that inoculation strategies and timing (i.e., simultaneous or sequential inoculation of LAB and yeasts) lead to the production of different aroma compounds modifying wine profile (Virdis et al., 2021). Moreover, the development of tailored starter cultures of LAB and yeasts are useful to minimize the sulfur dose (Nardi, 2020). For instance, *T. delbrueckii* has been proposed as an alternative to the use of SO_2_ if inoculated at the beginning of the white winemaking process (Simonin et al., 2018).

## Polyphenols Adsorption and Yeast Cell Wall

**FIGURE 1:**
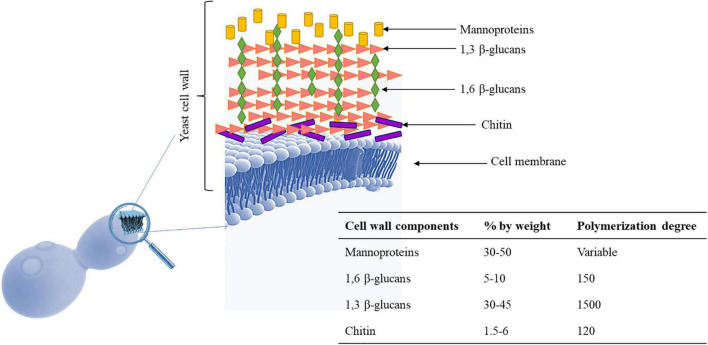
Yeast cell wall. It is made up of mannoproteins, β-1,3-glucans, β-1,6-glucans, and chitin. Cell wall may undergo several changes during alcoholic fermentation because they are exposed to several stresses (osmotic stress, low pH, high acidity, nitrogen depletion, elevated ethanol) which should increase anthocyanins adsorption. Macromolecules of *S. cerevisiae* cell wall (Klis et al., 2006; De Iseppi et al., 2020).

**FIGURE 2:**
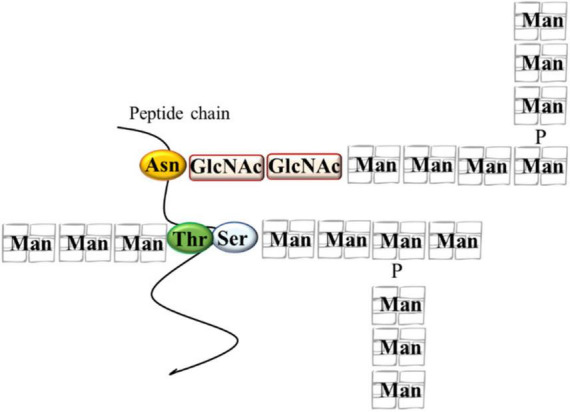
Some structural characteristics of mannoproteins. Asn, asparagine; GlcNAc, N-acetylglucosamine; Man, mannose; P, phosphate; Ser, serine; Thr, threonine.

The main role of the yeast cell wall is conferring protection and resistance to environmental conditions. The *S. cerevisiae* cell wall is 100–150 nm thick representing 10–25% of cell dry mass (Yin et al., 2007) and has a bi-layered structure. The outer layer of about 30–40 nm thick is mainly composed of mannoproteins covalently linked to the underlying glycans. The inner layer of about 70–100 nm consisting of a network of branched β-glucans (mainly β-1,3 glucans), serving as a scaffold for the entire cell wall and chitin molecules (Klis et al., 2006; Figure 1). Mannoproteins (MPs), β-1,3 glucans, β-1,6 glucans, and chitin, four polysaccharides that are covalently joined, constitute the structure of wall (Schiavone et al., 2017). Highly glycosylated mannoproteins constitute yeast mannan, a complex oligosaccharide comprising 10 to more than 50 mannose units linked in α-(1,2), α-(1,3), α-(1,5), and α-(1,6), which is attached to proteins by either Asn (large manno-oligosaccharides for N-glycosylation) or Ser/Thr residues (short manno- oligosaccharides to make the O-glycosylation) (Figure 2). Linear chains of about 1,500 glucose units linked in β-1,3 and β-1,6 compose the β-glucan, whereas 140–350 glucose units linked in β-1,6 form glucan. Chitin is a polymer 100–190 *N*-acetylglucosamine units linked by β-1,4 linkages (for reviews see Lesage and Bussey, 2006; Francois, 2016). The dry mass of cell wall is made of 50% of β-glucan, 40% of mannans, 3–5% of chitin and then proteins (Schiavone et al., 2014). These components are assembled each other to form a supramolecular architecture, cross-linked in various ways to form higher-order complexes. A central role in this cross-linking is carried out by β-1,6 glucan, even if it is a minor cell wall component from a quantitative point of view (Kollár et al., 1997). The cross-linking cell wall protein (CWPs) to β-1,3 glucans is carried out by β-1,6 glucans in connection with the glycosylphosphatidyl inositol (GPI) anchor attached to these proteins. Proteins with internal repeats (PIR)-CWPs are cell wall proteins directly linked to β-1,3 glucans through γ-carboxylic group of glutamates (Cabib et al., 2012). Cell wall composition varies over the yeast species and strains (Nguyen et al., 1998). Yeast cell wall proteins contain several tandem repeats, which vary greatly in number. Mutations in such repeats are associated to a great functional diversity, which allow yeasts to adapt to different ecological niches or facilitating their exploration of new ones (Verstrepen and Fink, 2009). The number of genes that encode enzymes directly involved in biosynthesis or remodeling of the wall, or non-enzymatic wall proteins, is about 200 genes. During growth and development yeast wall composition and degree of cross-linking can vary (Francois, 2016). Four functions have been recognized for the cell wall, namely stabilization and internal osmotic conditions, protection against stresses, maintenance of the cell shape and integrity, and a scaffold for cell wall proteins (Klis et al., 2006). These functions can be influenced by different factors such as single-strain characteristic, fermentation processes, chemical and environmental stress, substrate composition, and others. The cell wall polysaccharides possess technological properties, relevant for different applications in food safety, biotechnology, and technology (reviewed in Chen and Seviour, 2007; Kogani et al., 2008; Braconi et al., 2011; Pfliegler et al., 2015). In winemaking the role of yeast cell wall components is of great interest for managing fermentations, wine stabilization and aging processes. Several studies recognized a key role of MPs in the determination of wine color. MPs are polysaccharides released by yeast cells during wine fermentation and during aging of wine on lees by endo-glucanases, exo-D-mannose, and α-D-mannosidase (Arévalo Villena et al., 2005; Belda et al., 2016; Balmaseda et al., 2021). These proteins are mainly composed of mannose and glucose with a protein content ranging between 1 and 10% with a molecular weight ranging from 50 to 500 kDa (Yue et al., 2021). MPs protect wine against protein precipitation and stabilize wine color intensity. In fact, yeast MPs can combine with anthocyanins and tannins increasing color stability (Escot et al., 2001). In fact, the addition of MPs before AF enhances the content of anthocyanins and phenolic acids improving the color stability (Yue et al., 2021) and could protect the degradation of phenolic acids during the fermentation process playing a protective role (Fernando et al., 2018; Rinaldi et al., 2019). However, Mekoue Nguela et al. (2019) reported that polyphenol adsorption on yeast cell outer surface can have negative consequences on the cell wall metabolic activity interfering with cell signaling functions and nutrient transport. Interactions between yeast and polyphenols have been observed in wine aging on lees, a practice applied after fermentation to maintain the wine in contact with dead yeast cells (lees) (Mazauric and Salmon, 2005, 2006). The MPs’ influence on wine color is also dependent upon the strain of yeast used (Escot et al., 2001). Even if *S. cerevisiae* cell wall is considered the main source of MPs, also some NS yeasts such as *Schizosaccharomyces pombe, Pichia fermentans, M. pulcherrima, Saccharomycodes ludwigii, T. delbrueckii, Lachancea thermotolerans*, and *Wickerhamomyces anomalus*, demonstrated the ability to produce and release MPs into the wine during aging on lees (Morata et al., 2006, 2019; Belda et al., 2016; Ferrando et al., 2020; Balmaseda et al., 2021). MPs are released continuously during the growth of several NS yeasts, reflecting a high production of these polysaccharides during the first phase of fermentation (Domizio et al., 2014). Some studies highlighted that NS yeasts showed a higher release of MPs in wine than *S. cerevisiae* (for a review see Vejarano, 2020) with *S’codes ludwigii* are found among the species with high potentials for releasing polysaccharides (Palomero et al., 2009; Domizio et al., 2017). According to Palomero et al. (2009) *S’codes ludwigii* released 110.51 mg/L of MPs against the 36.65 mg/L of *S. cerevisiae*. non-*Saccharomyces* MPs showed a different structure in terms of protein, mannose, glucose, and galactose content compared to those characterizing *S. cerevisiae*. For instance, the % of mannose residues is 88% in *S. cerevisiae*, while range from 55% in *Sch. pombe* to 93% in *S’codes ludwigii.* Moreover, α-galactomannose rather than mannose has been found as part of the structure of polysaccharides in *Sch. pombe*. The polysaccharides from these NS yeasts show a greater molecular size and may potentially impact the wine’s palatability (Vejarano, 2020).

During aging on lees the color loss can occur since lees can interact with anthocyanins through pigments adsorption by lees and anthocyanins degradation by β-glucosidase enzymes (Darriet et al., 2012). Lees from different yeasts can have a different adsorption. For instance, lees from *M. pulcherrima*, *S’codes ludwigii*, or *Sch. pombe* have shown a low adsorption of anthocyanins with respect to the lees of *S. cerevisiae, T. delbrueckii*, or *L. thermotolerans* (Herderich et al., 2013). The less color loss could be also related to the ability of these NS yeasts, especially, *S. pombe*, to produce pyranoanthocyanins which are more stable than anthocyanins (Claus and Mojsov, 2018). In addition, *S. pombe*—besides its deacidification activity due to its ability to convert malic acid into ethanol and carbon dioxide—is able to improve color stability of red wine producing vitisin A and anthocyanin-vinyl phenol derivatives thanks to its capacity to release pyruvic acid and hydroxycinnamate decarboxylase activity (Morata et al., 2012).

## Influence of Yeasts on Polyphenolic Profile of Wines

Wines are characterized by a certain variety of phenolic compounds also known as polyphenols or biophenols. Grape polyphenols are secondary compounds extracted during the winemaking process which contribute to wine color and flavor especially in red wines (Goldner and Zamora, 2007). In fact, in red wine their concentration is approximately about six times higher than that in white one because red juice has longer contact time with the grape skins and seeds. In particular, the minimum and maximum levels of total phenolic contents reported (expressed in mg of gallic acid equivalents per liter) ranged from 1,531 to 3,192 and from 210 to 402 for red and white wines, respectively (Visioli et al., 2020). They are composed of one or more hydroxyl groups linked with one or more aromatic or benzene rings (Visioli et al., 2020). Moreover, these compounds can be conjugated to one or more sugar residues linked by β-glycosidic (*O*-glycosylated) bonds or by direct linkages of sugar to an aromatic ring carbon atom (*C*-glycosides) (Visioli et al., 2020). They can be classified into 2 groups: the flavonoids and the non-flavonoids. The first one includes anhocyanins, flavonols, and flavonoids. The second one encompasses hydroxybenzoic acids, hydroxycinnamates, and the stilbenoids (Jaganath and Crozier, 2010; Table 2). The concentration of polyphenols in wines is influenced by viticulture (grape variety and clone, light exposure, degree of ripeness), vinification process (destemming, crushing, pre-fermentation maceration, alcoholic fermentation, pressing), and yeast strains (Jagatić Korenika et al., 2021). For instance, must freezing, cryogenic maceration, extended maceration, and temperature increase phenolics concentrations in wines, while mechanical harvesting could decrease their concentration through reactions with oxidative radicals (Olejar et al., 2015).

**TABLE 2:** Characteristics of main polyphenols occurring in wine (modified by Visioli et al., 2020).

Polyphenols	Characteristics	
**Flavonoids**		
Anthocyanins	They are water-soluble pigments and the main anthocyanins in wines are anthocyanidin−3-*O-*glucosides, peonidin-3-glc, cyanidin-3-glc, petunidin-3-glc, and delphinidin−3-glc. Their concentration can reach 400 mg/L in red wines, while are absent in white ones. Their effect on wine color is influenced by several factors and the main are pH and co-pigmentation. At low pH the red color is stable, while in presence of alkaline conditions they appear purple/blue. Anthocyanins can interact with other polyphenols (co-pigmentation) stabilizing wine color. In particular, during fermentation and aging, anthocyanins are chemically modified by their interaction with pyruvic acid, coumaric acid, ethanal, flavan-3-ols, condensed tannins, and other reactive molecules yielding pyroanthocyanins and polymerized pigments	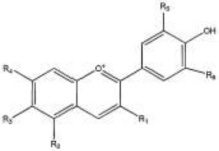
Flavan-3-ol	They are yellow pigments responsible of wines’ astringency, bitterness, and structure and can be found in monomeric form (catechin and epicatechin) and in their polymeric form (proanthocyanidins, also called condensed or non-hydrolysable tannins). The main ones detected in grapes and wine are myricetin, quercetin, laricitrin, kaempferol, isorhamnetin, and syringetin. They can be found in both white and red wines with values ranging from 15 to 25 mg/L and from 4 to 120 mg/L, respectively.	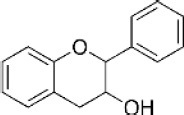
Flavonoids	They exist as glycosides in combination with monosaccharides such as glucose, rhamnose, galactose, xylose, and arabinose.	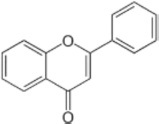
**Non-flavonoids**		
Hydroxybenzoic acids	The most abundant are p-hydroxybenzoic, gallic, vanillic, gentisic, syringic, salicylic, and protocatechuic acids. The gallic acid has been found in red and white wines with concentrations 70 and 10 mg/L, respectively.	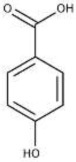
Hydroxycinnamic acids	Hydroxycinnamic acids are the main group of polyphenols in must and white wine. They are generally conjugated with tartaric acid esters or diesters and responsible of wine browning processes since they can be oxidized.	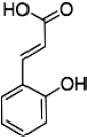
Stilbenes	The main stilbenes found in wines are trans-piceid and trans-resveratrol, hopeaphenol, ampelosin A, isohopeaphenol, piceatannol, pallidol, *e*-viniferin, miyabenol C, *r*-viniferin, and *r2*-viniferin. In general, they occur in low concentrations, but if grapes are subjected to abiotic and biotic stresses the amount of resveratrol can reach values of 100 mg/L.	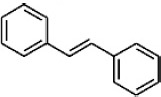

The first evidence of yeast influence on polyphenols content was reported by Caridi et al. (2004) who highlighted the correlations between yeast strain and chromatic properties, and phenolic profile of wines. Subsequently, Medina et al. (2005) reported the importance of yeast selection to shape anthocyanin concentration and Morata et al. (2016) demonstrated that yeast strains are involved in stable pigments formation and in the adsorption of color molecules on the cell wall. In fact, yeast metabolism may lead to different values of metabolic precursors during must fermentation for the formation of pyranoanthocyanins, oligomeric, and polymeric pigments (Escott et al., 2018).

Actually, 3 different mechanisms have been described to explain the interaction between the wine’s polyphenols and yeasts (Giovinazzo et al., 2019). The first one is based on the adsorption of polyphenols on the yeast cell wall. This phenomenon is strain dependent and not yet completely understood. It probably depends on cell wall surface structure and composition being apolar anthocyanins better adsorbed than polar ones. According to Echeverrigaray et al. (2019), yeasts could be grouped as low, medium, and high anthocyanins adsorption strains. The same authors demonstrated that yeast anthocyanin adsorption occurs by pigment-binding molecules constitutively expressed in the inner part of the cell walls of all *Saccharomyces* strains, regardless of their assigned adsorption behavior in red wine fermentation (high, medium, and low pigment adsorption yeast strains) (Echeverrigaray et al., 2020). Recent studies showed that yeast cells primarily adsorb grape pigments by the end of fermentation (Echeverrigaray et al., 2019, 2020). The second mechanism is linked to yeast β-glucosidase activity, which breaks the polyphenols–sugar bond, anthocyanidase enzymes, or pectinolytic enzymes which favor the extraction of color from pomace (Manzanares et al., 2000). Glycosidase activities have been described in various NS yeasts (*Candida, Hanseniaspora, Pichia, Metschnikowia, Rhodotorula, Trichosporon, Wickerhamomyces*) (for a review see Claus and Mojsov, 2018). Hydrolysis of glucose usually results in a corresponding anthocyanidin, which is converted to the colorless pseudobase, which may affect color and stability (Mansfield et al., 2002). The last one is based on the release by yeast strains of polysaccharides, like MPs, able to entrap polyphenols during fermentation (see previous paragraph).

**FIGURE 3:**
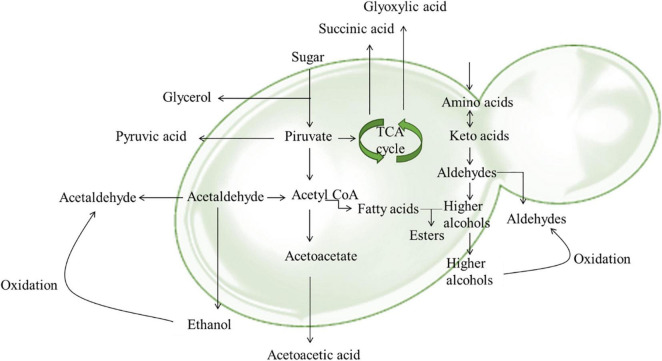
Main pathways involved in the formation of anthocyanin derivatives and polymeric pigments.

Of particular interest for the determination of wine color is the adsorption of anthocyanins. During fermentation and aging, anthocyanins are subjected to chemical modifications through their interaction with other compounds including pyruvic acid, flavan-3-ols, condensed tannins, etc. (Morata et al., 2016; Figure 3). These modifications together with structural or metabolic modifications of yeast increase their adsorbability by yeast cell wall. These changes have been found to occur during alcoholic fermentation as a response to stresses such as high osmotic pressure, low pH, high acidity, nitrogen depletion, elevated ethanol, in order to maintain metabolic activity and cell viability (Klis et al., 2002; Aguilar-Uscanga and François, 2003; Duc et al., 2017). Yeast stress response depend on several genes and varies among yeasts strains winemaking conditions (Stanley et al., 2010; Duc et al., 2017). In general, cell viability and cell wall integrity are negatively correlated with pigment adsorption and induce the phenotypic differences among strains (Echeverrigaray et al., 2020; for a review see Zhang et al., 2021).

Morata et al. (2016) showed that adsorption mechanisms can be responsible for up to 6% reduction of anthocyanins that contribute to wine color intensity, significantly impacting the quality of the final product. Yeast adsorption and its impact on wine color has been corroborated by multiple studies (Morata et al., 2003, 2016; Medina et al., 2005; Caridi et al., 2007), and this trait has been identified as a target for yeast strain selection toward the improvement for wine production. The importance of yeast strain selection has been shown also by Carew et al. (2013) who demonstrated a significant influence of yeast strain on the concentration and composition of wine tannins. The use of *S. cerivisiae* RC212 results in wines with high concentration of total pigment, free anthocyanin, non-bleachable pigment, and total tannin, and showed high color density. Moreover, the sequential inoculation of *S. cerivisiae* RC212 and *T. delbruekii* allowed to obtain wines with a high degree of tannin polymerization. Different behavior of two *S. cerevisiae* strains, and a *S. bayanus* strain on the phenolic profile of Aurora white wine have been reported by Samoticha et al. (2019). *S. cerevisiae* strains produced higher amounts of polyphenols. In fact, resulting wines had a content of total polyphenols of about 300 mg/L and a high antioxidant capacity. Similarly, Grieco et al. (2019) highlighted the importance to select autochthonous tailored yeast strains to modulate the phenolic composition of Negroamaro and Primitivo wines. A recent study revealed that the use of *M. pulcherrima*, *Z. bailii*, *Candida zeylanoides*, and *T. delbrueckii* increased the content of monomeric anthocyanin in Tempranillo wines improving their color and health properties (Escribano-Viana et al., 2019). Similar results concerning *T. delbrueckii* were obtained by Balmaseda et al. (2021). These authors revealed that the use of *T. delbrueckii* improved the volatile complexity and polyphenolic composition of wines and enabled spontaneous MLF. Moreover, some yeast metabolites can react with anthocyanins forming more stable pigments. Visitin A is formed during the fermentation process and aging from pyruvic acid produced by yeasts and malvidin-3-*O*-glucoside, while visitin B from malvidin-3-*O*-glucoside and acetaldehyde (Morata et al., 2003).

Pyruvate is produced during the catabolism of sugars and can be metabolized into acetaldehyde, or used in the formation of acetyl-CoA (Morata et al., 2003). Acetaldehyde is a byproduct of yeast metabolism, and can be also produced through a non-enzymatic oxidation of ethanol (Danilewicz, 2003). Acetaldehyde concentrations increase during the fermentation process and as a wine is exposed to oxygen. A controlled oxidation of wine is highly recommended since uncontrolled introduction of oxygen can cause alterations of wine color, and the loss of desirable aromas and formation of undesirable aromas, and even promote aerobic bacteria (Gómez-Plaza and Cano-López, 2011). Generally, acetaldehyde reacts with sulfur dioxide (SO_2_), or with other compounds such as tannins influencing wine color (Carlton et al., 2007). In fact, it promotes rapid polymerization between anthocyanins and catechins or tannins, forming stable polymeric pigments resistant to SO_2_ bleaching (Timberlake and Bridle, 1976).

Acetaldehyde and pyruvic acid production are strain specific and is particularly evident in NS yeasts. For instance, *Sch. pombe* released a higher concentration of pyruvate than *S. cerevisiae* during fermentation (Morata et al., 2012; Belda et al., 2017). *Torulaspora delbrueckii* produces low amount of acetaldehyde, in comparison with *S. cerevisiae*. This trait is interesting not only in terms of wine color because is related to visitin B production, but also since concentrations above 125 mg/L of acetaldehyde has a negative effect on wine’s flavor (Benito et al., 2019). Therefore, the selection NS yeast with a suitable production of pyruvate and acetaldehyde to be used in combination with *S. cerevisiae* could represent a useful strategy to increase visitins production during must fermentation. Some NS yeasts can release up to four times higher concentrations of pyruvic acid or acetaldehyde than *S. cerevisiae*. The combination of tailored NS species/strains could allow the microbial stabilization of wines, avoiding malolactic fermentation and increase the acidity and color perception (Chen et al., 2018; Benito et al., 2019). Moreover, *S. cerevisiae* as well as NS yeasts have hydroxycinnamate decarboxylase (HCDC) activity and are able to produce vinylphenolic pyranoanthocyanins (VPAs) from the chemical interaction between hydroxycinnamic acids and anthocyanins (Morata et al., 2007). Hydroxycinnamic acids could also directly react with anthocyanins and form VPAs without enzymatic support (Schwarz et al., 2003). HCDC activity has been described in several yeast species (Morata et al., 2012). Recently, this activity was tested in 14 different yeast genera (*Wickerhamomyces*, *Torulaspora*, *Starmerella*, *Pichia*, *Metschnikowia*, *Lachancea*, *Kregervanrija*, *Kluyveromyces*, *Kodamaea*, *Issatchenkia*, *Hanseniaspora*, *Debaryomyces*, *Candida*, *Meyerozyma*) and revealed that *M. guilliermondii* and *W. anomalus* strains had the highest HCDC activity, while *S. servazii*, *M. fructicola*, *K. dobzhanskii*, *H. osmophila*, *C. sake* strains the lowest (Božič et al., 2020). Moreover, Escott et al. (2018) found a higher concentration of stable pigments produced during fermentation with non-*Saccharomyces* yeasts in comparison to pure fermentations with *S. cerevisiae*.

Independent of their adsorption behavior during red wine fermentation, damaged yeast cells showed the same anthocyanin adsorption capacity, indicating that any major differences in anthocyanin adsorption between yeast strains are determined by their ability to maintain cell viability, as well as the cell wall and membrane integrity throughout wine fermentation (Echeverrigaray et al., 2020). Moreover, these results suggest that anthocyanin adsorption binding molecules, probably MPs, did not vary significantly among strains, and that such factors are localized in the inner part of yeast cell walls, as previously suggested by observations by Vasserot et al. (1997), Petruzzi et al. (2015), and Gonçales et al. (2018).

Studies carried out some years ago found that some *S. cerevisiae* strains could stabilize white wine color after exposure to air and light (Suzzi et al., 1985). This ability called “stabilizing power” varied in relation to sulfite production; low sulfite forming strains did not produce stable wines, whereas strains able to stabilize wine color were high sulfite forming ones. However, a direct relationship between SO_2_ produced during fermentation and stabilization ability was not always recorded (for a review see Romano and Suzzi, 1993).

Yeasts can also influence wine color releasing organic acids. Englezos et al. (2018) revealed that *Starm. bacillaris* produced pyruvic acid, acting as a natural acidification agent by reducing the wine’s pH. The acidogenic nature of *Starm. bacillaris* could have an impact on wine color stability, mainly due to the ability of the pyruvic acid to bind sulfur dioxide and swift the equilibrium of anthocyanins from the colorless to colored form due to the reaction of pyruvic acid with anthocyanins producing stable pigments such as vitisin A (Englezos et al., 2018).

Some studies also focused on the role of flor yeast to decrease browning in white wines (Fabios et al., 2000; Merida et al., 2005). *Saccharomyces cerevisiae* flor yeast or flor velum yeasts can grow at the surface of different wines are involved in their biological aging (David-Vaizant and Alexandre, 2018). During biological aging these yeasts shifts from a fermentative to an oxidative metabolism (diauxic shift) induced by nitrogen and sugar depletion and rise to the wine surface to form multicellular aggregates. This aggregation leads to the build-up of a biofilm, or velum or flor (Legras et al., 2016). Biofilm formation begins under nutrients starvation and is favored by the presence of other carbon sources, such as glycerol and ethyl acetate (Zara et al., 2010). Flor yeasts are able to face wine stresses characterizing mainly induced by ethanol and acetaldehyde (Zara et al., 2010). This strong adaptation is probably related to DNA mutations responsible for mitochondrial DNA polymorphism and chromosomal rearrangements (Legras et al., 2016). Some studies suggested that probably these yeasts are able to protect wine from browning since they consume the oxygen through their aerobic metabolism and because they retain brown pigments on the cell wall. Some authors reported in presence of flor yeasts a gradual disappearance of catechin. This phenomenon may be due to the production of acetaldehyde during flor yeasts aerobic growth (Cortes et al., 1998), thereby favoring the formation of oligomers (Fulcrand et al., 1998).

## Influence of Lactic Acid Bacteria on Wine Color

**FIGURE 4:**
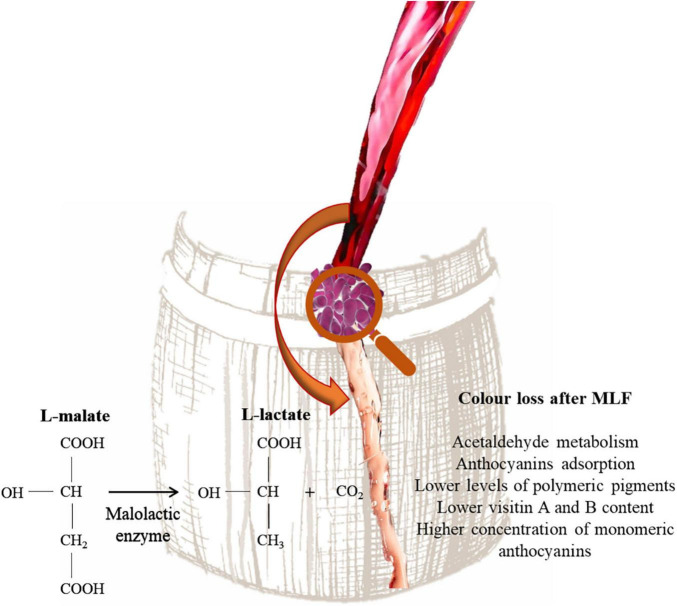
Influence of MLF on wine color.

Lactic acid bacteria are responsible of MLF. During this process L-malic acid is converted into L-lactic acid and reduce the acidity of wine. The consumption of L-malic acid reduces the risk of the wine spoilage and improve the palatability of wine (Sumby et al., 2019). In addition, aroma precursors in wine can be further hydrolyzed into free-form volatiles under malolactic fermentation (Lonvaud-Funel, 1999). Color loss is common in wines that have undergone MLF (Burns and Osborne, 2013). In fact, these wines, independently from pH, are characterized by lower levels of polymeric pigments, lower visitin A and B content and a higher concentration of monomeric anthocyanins than wines that did not undergo MLF (Figure 4). This phenomenon has been explained by LAB ability to utilize acetaldehyde and pyruvic acid during MLF (Devi and Ka, 2019). This compound is essential for ethylene-linked pigments formation, which are more stable than their respective monomeric anthocyanins and show better colorimetric properties (Forino et al., 2020). Moreover, pyruvic acid can react with malvidin-3-glucoside, forming pyranoanthocyanins e.g., visitin A and visitin B (Waterhouse et al., 2016). Moreover, Devi et al. (2019) showed that *O. oeni* and *Lactiplantibacillus plantarum* strains are able to adsorb delphinidin-3-glucoside, malvidin-3-glucoside and peonidin-3-glucoside on the cell wall and can also produce β-glycosidase enzymes. However, it has been reported that some LAB strains belonging to *Lactiplantibacillus, Leuconostoc, Pediococcus*, and *Streptococcus* genera are able to produce acetaldehyde (Liu and Pilone, 2000). Wang et al. (2018) showed that *Lpb. plantarum* inoculated wine after MLF showed higher level of pyranoanthocyanins, whereas the use of *O. oeni* strains resulted in less formation of pyranoanthocyanins in wine. Moreover, *Lpb. plantarum* strains increased the accumulation of acetaldehyde in wine model medium and wine during malolactic fermentation. The influence on wine color depends also on the yeast/bacteria inoculation strategy. In fact, when MLF is performed with sequential inoculation a higher color loss is observed (Devi and Ka, 2019). Actually, some studies are focused on the long-term effects of MLF on wine color. Izquierdo-Cañas et al. (2016) reported that 9 months after the end of MLF resulting in color intensity loss and lower acylated and non-acylated anthocyanins levels. However, an increase of the pyranoanthocyanin concentration was observed.

## Conclusion

Wine characteristics depend on grape berry phenolic composition, and on microbial activities during fermentation. Yeasts belonging to the genus *Saccharomyces* are considered the main actors of wine fermentation. However, non-*Saccharomyces* yeasts can be exploited as potential starters in mixed fermentations with *S*. *cerevisiae.* The understanding and managing of yeasts, their diversity and effects on wine quality can be optimized, resulting in better organoleptic characteristics, such as color and aroma. Yeasts can impact wine color through at least 3 different mechanisms: (i) release of metabolites which could participate in the red wine color stabilization process and increase the content of stable pigments; (ii) presence of enzymatic activities such as glycosidase and pectinase; (iii) adsorption of phenolic compounds by yeast cell wall, especially anthocyanins and tannins, which largely leads to the loss of red wine color and reduction of astringency. Different strains of *S. cerevisiae* and NS yeasts have been found to influence wine color in a different way, mostly due to their variations in the forementioned three mechanisms. In this sense population studies might be useful. In fact, the ratio of low and high adsorbing cell populations varied among wine yeast strains, and is related to yeast fermentative life-span or cell viability. Tailored yeast strains can affect and stabilize wine color and pigments. The wide diversity of effects on polyphenols and on the final wine color in single and mixed fermentations carried out by *S. cerevisiae* and NS strains indicates the great importance of these studies for the future of winemaking. Moreover, the influence of LAB on wine color should be also considered. A key point is the establishment of the right time for promoting MLF to prevent consumption of pyruvic acid by LAB and to promote vitisin synthesis. Some yeast-derived compounds such as mannoproteins can be stimulatory for *O. oeni.* Further studies are necessary to clarify the regulation of mannoprotein metabolism in LAB and to evaluate the effect of mannoproteins released by different yeasts on LAB fitness and MLF kinetics. Moreover, a better knowledge on yeasts/bacteria interactions during fermentation and on the effects of inoculation strategies should be achieved to improve wine color and contribute to consumers’ purchasing decision.

## Author Contributions

RT: conceptualization, writing—review and editing, and funding acquisition. GS: review and editing. GP: writing—original draft and writing—review and editing. All authors contributed to the article and approved the submitted version.

## Conflict of Interest

The authors declare that the research was conducted in the absence of any commercial or financial relationships that could be construed as a potential conflict of interest.

## Publisher’s Note

All claims expressed in this article are solely those of the authors and do not necessarily represent those of their affiliated organizations, or those of the publisher, the editors and the reviewers. Any product that may be evaluated in this article, or claim that may be made by its manufacturer, is not guaranteed or endorsed by the publisher.

## References

[B1] Aguilar-Uscanga B. François J. M. (2003). A study of the yeast cell wall composition and structure in response to growth conditions and mode of cultivation. *Lett. Appl. Microbiol.* 37 268–274. 10.1046/j.1472-765X.2003.01394.x 12904232

[B2] Álvarez-Pérez J. M. Campo E. San-Juan F. Coque J. J. R. Ferreira V. Hernández-Orte P. (2012). Sensory and chemical characterisation of the aroma of prieto picudo rosé wines: the differential role of autochthonous yeast strains on aroma profiles. *Food Chem.* 133 284–292. 10.1016/j.foodchem.2012.01.024 25683397

[B3] Arévalo Villena M. Úbeda Iranzo J. Cordero Otero R. Briones Pérez A. (2005). Optimization of a rapid method for studying the cellular location of β-glucosidase activity in wine yeasts. *J. Appl. Microbiol.* 99 558–564. 10.1111/j.1365-2672.2005.02627.x 16108797

[B4] Balmaseda A. Aniballi L. Rozès N. Bordons A. Reguant C. (2021). Use of yeast mannoproteins by *Oenococcus oeni* during malolactic fermentation under different oenological conditions. *Foods* 10:1540. 10.3390/foods10071540 34359413 PMC8305826

[B5] Barbosa C. Lage P. Esteves M. Chambel L. Mendes-Faia A. Mendes-Ferreira A. (2018). Molecular and phenotypic characterization of *Metschnikowia pulcherrima* strains from Douro Wine Region. *Fermentation* 4:8. 10.3390/fermentation4010008

[B6] Belda I. Navascués E. Marquina D. Santos A. Calderón F. Benito S. (2016). Outlining the influence of non-conventional yeasts in wine ageing over lees. *Yeast* 33 329–338. 10.1002/yea.3165 27017923

[B7] Belda I. Ruiz J. Beisert B. Navascués E. Marquina D. Calderón F. (2017). Influence of *Torulaspora delbrueckii* in varietal thiol (3-SH and 4-MSP) release in wine sequential fermentations. *Int. J. Food Microbiol.* 257 183–191. 10.1016/j.ijfoodmicro.2017.06.028 28668728

[B8] Bellon J. R. Schmid F. Capone D. L. Dunn B. L. Chambers P. J. (2013). Introducing a new breed of wine yeast: interspecific hybridisation between a commercial *Saccharomyces cerevisiae* wine yeast and *Saccharomyces mikatae*. *PLoS One* 8:e62053. 10.1371/journal.pone.0062053 23614011 PMC3629166

[B9] Bely M. Stoeckle P. Masneuf-Pomarède I. Dubourdieu D. (2008). Impact of mixed *Torulaspora delbrueckii-Saccharomyces cerevisiae* culture on high-sugar fermentation. *Int. J. Food Microbiol.* 122 312–320. 10.1016/j.ijfoodmicro.2007.12.023 18262301

[B10] Benito Á Calderón F. Benito S. (2019). The influence of non-*Saccharomyces* species on wine fermentation quality 540 parameters. *Fermentation* 5 1–18. 10.3390/fermentation5030054

[B11] Benito Á Calderón F. Palomero F. Benito S. (2016). Quality and composition of airén wines fermented by sequential inoculation of *Lachancea thermotolerans* and *Saccharomyces cerevisiae*. *Food Technol. Biotechnol.* 54 135–144. 10.17113/ftb.54.02.16.4220 27904403 PMC5105610

[B12] Benito S. (2018). The impact of *Torulaspora delbrueckii* yeast in winemaking. *Appl. Microbiol. Biotechnol.* 102 3081–3094. 10.1007/s00253-018-8849-0 29492641

[B13] Benito S. Palomero F. Calderón F. Palmero D. Suárez-Lepe J. A. (2014). Selection of appropriate *Schizosaccharomyces* strains for winemaking. *Food Microbiol.* 42 218–224. 10.1016/j.fm.2014.03.014 24929740

[B14] Benito S. Palomero F. Morata A. Calderón F. Suárez-Lepe J. A. (2012). New applications for *Schizosaccharomyces pombe* in the alcoholic fermentation of red wines. *Int. J. Food Sci. Technol.* 47 2101–2108. 10.1111/j.1365-2621.2012.03076.x

[B15] Bokulich N. A. Ohta M. Richardson P. M. Mills D. A. (2013). Monitoring seasonal changes in winery-resident microbiota. *PLoS One* 8:e66437. 10.1371/journal.pone.0066437 23840468 PMC3686677

[B16] Božič J. T. Butinar L. Albreht A. Vovk I. Korte D. Vodopivec B. M. (2020). The impact of *Saccharomyces* and non-*Saccharomyces* yeasts on wine colour: a laboratory study of vinylphenolic pyranoanthocyanin formation and anthocyanin cell wall adsorption. *LWT Food Sci. Technol.* 123:109072. 10.1016/j.lwt.2020.109072

[B17] Braconi D. Amato L. Bernardini G. Arena S. Orlandini M. Scaloni A. (2011). Surfome analysis of a wild-type wine Saccharomyces cerevisiae strain. *Food Microbiol.* 28 1220–1230. 10.1016/j.fm.2011.04.009 21645823

[B18] Burns T. R. Osborne J. P. (2013). Impact of malolactic fermentation on the color and color stability of Pinot noir and Merlot wine. *Am. J. Enol. Vitic.* 64 370–377. 10.5344/ajev.2013.13001

[B19] Cabib E. Blanco N. Arroyo J. (2012). Presence of a large beta (1- 3) glucan linked to chitin at the Saccharomyces cerevisiae mother-bud neck suggests involvement in localized growth control. *Eukaryot. Cell* 11 388–400. 10.1128/EC.05328-11 22366124 PMC3318300

[B20] Carew A. L. Smith P. Close D. C. Curtin C. Dambergs R. G. (2013). Yeast effects on pinot noir wine phenolics, color, and tannin composition. *J. Agric. Food Chem.* 61 9892–9898. 10.1021/jf4018806 24011384

[B21] Caridi A. Cufari A. Lovino R. Palumbo R. Tedesco I. (2004). Influence of yeast on polyphenol composition of wine. *Food Technol. Biotechnol.* 42 37–40.

[B22] Caridi A. Sidari R. Solieri L. Cufari A. Giudici P. (2007). Wine colour adsorption phenotype: an inheritable quantitative trait loci of yeasts. *J. Appl. Microbiol.* 103 735–742. 10.1111/j.1365-2672.2007.03301.x 17714407

[B23] Carlton W. K. Gump B. Fugelsang K. Hasson A. S. (2007). Monitoring acetaldehyde concentrations during micro-oxygenation of red wine by headspace solid-phase microextraction with on-fiber derivatization. *J. Agric. Food Chem.* 55 5620–5625. 10.1021/jf070243b 17567026

[B24] Chen J. Seviour R. (2007). Medicinal importance of fungal beta-(1–>3), (1–>6)-glucans. *Mycol. Res.* 111 635–652. 10.1016/j.mycres.2007.02.011 17590323

[B25] Chen K. Escott C. Loira I. del Fresno J. M. Morata A. Tesfaye W. (2018). Use of non-*Saccharomyces yeasts* and oenological tannin in red winemaking: influence on colour, aroma and sensorial properties of young wines. *Food Microbiol.* 69 51–63. 10.1016/j.fm.2017.07.018 28941909

[B26] Claus H. Mojsov K. (2018). Enzymes for wine fermentation: current and perspective applications. *Fermentation* 4:52. 10.3390/fermentation4030052

[B27] Comitini F. Gobbi M. Domizio P. Romani C. Lencioni L. Mannazzu I. (2011). Selected non-*Saccharomyces* wine yeasts in controlled multistarter fermentations with *Saccharomyces cerevisiae*. *Food Microbiol.* 28 873–882. 10.1016/j.fm.2010.12.001 21569929

[B28] Cortes M. B. Moreno J. Zea L. Moyano L. Medina M. (1998). Changes in aroma compounds of Sherry wines during their biological aging carried out by *Saccharomyces cerevisiae* races bayanus and capensis. *J. Agric. Food Chem.* 46 2389–2394. 10.1021/jf970903k

[B29] Danilewicz J. C. (2003). Review of reaction mechanisms of oxygen and proposed intermediate reaction products in wine: central role of iron and copper. *Am. J. Enol. Vitic.* 54 73–85.

[B30] Darriet P. Thibon C. Dubourdieu D. (2012). “Aroma and aroma precursors in grape berry,” in *Aroma and Aroma Precursors in Grape Berry*, eds Hernâni Gerós M. Manuela C. Serge Bentham D. (Sharjah: Science Publishers). 10.2174/978160805360511201010111

[B31] David-Vaizant V. Alexandre H. (2018). Flor Yeast diversity and dynamics in biologically aged wines. *Front. Microbiol.* 9:2235. 10.3389/fmicb.2018.02235 30319565 PMC6167421

[B32] De Iseppi A. Lomolino G. Marangon M. Curioni A. (2020). Current and future strategies for wine yeast lees valorization. *Food Res. Int.* 137:109352. 10.1016/j.foodres.2020.109352 33233056

[B33] Devi A. Aiyappaa A. A. K. Waterhouse A. L. (2019). Adsorption and biotransformation of anthocyanin glucosides and quercetin glycosides by *Oenococcus oeni* and *Lactobacillus plantarum* in model wine solution. *J. Sci. Food. Agric.* 100 2110–2120. 10.1002/jsfa.10234 31875958

[B34] Devi A. Ka A.-A. (2019). Yeast-bacterial interactions during malolactic inoculations affecting anthocyanin adsorption and content in Shiraz wine. *Am. J. Enol. Vitic.* 71 105–112. 10.5344/ajev.2019.19033

[B35] Domizio P. Lencioni L. Calamai L. Portaro L. Bisson L. F. (2018). Evaluation of the yeast Schizosaccharomyces japonicus for use in wine production. *Am. J. Enol. Vitic.* 69 266–277. 10.5344/ajev.2018.18004

[B36] Domizio P. Liu Y. Bisson L. F. Barile D. (2014). Use of non-Saccharomyces wine yeasts as novel sources of mannoproteins in wine. *Food Microbiol.* 43 5–15. 10.1016/j.fm.2014.04.005 24929876

[B37] Domizio P. Liu Y. Bisson L. F. Barile D. (2017). Cell wall polysaccharides released during the alcoholic fermentation by *Schizosaccharomyces pombe* and *S. japonicus*: quantification and characterization. *Food Microbiol.* 61 136–149. 10.1016/j.fm.2016.08.010 27697163 PMC5113737

[B38] Duc C. Pradal M. Sanchez I. Noble J. Tesnière C. Blondin B. (2017). A set of nutrient limitations trigger yeast cell death in a nitrogen-dependent manner during wine alcoholic fermentation. *PLoS One* 12:e0184838. 10.1371/journal.pone.0184838 28922393 PMC5602661

[B39] Echeverrigaray S. Menegotto M. Longaray Delamare A. P. (2019). A simple and reliable method for the quantitative evaluation of anthocyanin adsorption by wine yeasts. *J. Microbiol. Methods* 157 88–92. 10.1016/j.mimet.2018.12.016 30576751

[B40] Echeverrigaray S. Scariot F. J. Menegotto M. Longaray Delamare A. P. (2020). Anthocyanin adsorption by *Saccharomyces cerevisiae* during wine fermentation is associated to the loss of yeast cell wall/membrane integrity. *Int. J. Food Microbiol.* 314:108383. 10.1016/j.ijfoodmicro.2019.108383 31698283

[B41] Englezos V. Rantsiou K. Cravero F. Torchio F. Giacosa S. Ortiz-Julien A. (2018). Volatile profiles and chromatic characteristics of red wines produced with *Starmerella bacillaris* and *Saccharomyces cerevisiae*. *Food Res. Int.* 109 298–309. 10.1016/j.foodres.2018.04.027 29803453

[B42] Escot S. Feuillat M. Dulau L. Charpentier C. (2001). Release of polysaccharides by yeast and the influence of polysaccharides on colour stability and wine astringency. *Aust. J. Grape Wine Res.* 7 153–159. 10.1111/j.1755-0238.2001.tb00204.x

[B43] Escott C. del Fresno J. M. Loira I. Morata A. Tesfaye W. González M. C. (2018). Formation of polymeric pigments in red wine through sequential fermentation of flavanol-enriched musts with non-*Saccharomyces* yeasts. *Food Chem.* 239 975–983. 10.1016/j.foodchem.2017.07.037 28873660

[B44] Escribano-Viana R. Portu J. Garijo P. López R. Santamaría P. López-Alfaro I. (2019). Effect of the sequential inoculation of non-*Saccharomyces*/*Saccharomyces* on the anthocyans and stilbenes composition of Tempranillo wines. *Front. Microbiol.* 10:773. 10.3389/fmicb.2019.00773 31024516 PMC6465580

[B45] Fabios M. Lopez-Toledano A. Mayen M. Merida J. Medina M. (2000). Phenolic compounds and browning in Sherry wines subjected to oxidativeand biological ageing. *J. Agric. Food Chem.* 48 2155–2159. 10.1021/jf9908502 10888514

[B46] Fernando J. G. Pedro A. R. F. Dulcineia F. W. Susana M. C. Silvia M. R. Manuel A. C. (2018). Interaction of wine mannoproteins and arabinogalactans with anthocyanins. *Food Chem.* 243 1–10. 29146314 10.1016/j.foodchem.2017.09.097

[B47] Ferrando N. Araque I. Ortís A. Thornes G. Bautista-Gallego J. Bordons A. (2020). Evaluating the effect of using non-Saccharomyces on Oenococcus oeni and wine malolactic fermentation. *Food Res. Int.* 138(Pt B), 109779. 10.1016/j.foodres.2020.109779 33288165

[B48] Forino M. Picariello L. Lopatriello A. Moio L. Gambuti A. (2020). New insights into the chemical bases of wine color evolution and stability: the key role of acetaldehyde. *Eur. Food Res. Technol.* 246 733–743. 10.1007/s00217-020-03442-x

[B49] Francois J. M. (2016). Cell surface interference with plasma membrane and transport processes in yeasts. *Adv. Exp. Med. Biol.* 892 11–31. 10.1007/978-3-319-25304-6_2 26721269

[B50] Fulcrand H. Benabdeljalil C. Rigaud J. Chenyier V. Moutounet M. (1998). A new class of wine pigments generated by reaction between pyruvic acid and grape anthocyanins. *Phytochemistry* 47 1401–1407. 10.1016/S0031-9422(97)00772-3 9611832

[B51] Giovinazzo G. Carluccio M. A. Grieco F. (2019). “Wine polyphenols and health,” in *Bioactive Molecules in Food—Reference Series in Phytochemistry*, eds Mérillon J. M. Ramawat K. G. (Basel: Springer). 10.1007/978-3-319-78030-6_81

[B52] Gobbi M. Comitini F. Domizio P. Romani C. Lencioni L. Mannazzu I. (2013). *Lachancea thermotolerans* and *Saccharomyces cerevisiae* in simultaneous and sequential co-fermentation: a strategy to enhance acidity and improve the overall quality of wine. *Food Microbiol.* 33 271–281. 10.1016/j.fm.2012.10.004 23200661

[B53] Goldner C. M. Zamora C. M. (2007). Sensory characterization of *Vitis vinifera* cv. Malbec wines from seven viticulture regions of Argentina. *J. Sens. Stud.* 22 520–532. 10.1111/j.1745-459X.2007.00123.x

[B54] Gómez-Plaza E. Cano-López M. (2011). A review on microoxygenation of red wines: claims, benefits and the underlying chemistry. *Food Chem.* 125 1131–1140. 10.1016/j.foodchem.2010.10.034

[B55] Gonçales F. J. Fernandes P. A. R. Wessel D. F. Cardoso S. M. Rocha S. M. Coimbra M. (2018). Interaction of wine mannoproteins and arabinogalactans with anthocyanins. *Food Chem.* 243 1–10. 10.1016/j.foodchem.2017.09.097 29146314

[B56] Gonzalez S. S. Barrio E. Gafner J. Querol A. (2006). Natural hybrids from *Saccharomyces cerevisiae*, *Saccharomyces bayanus* and *Saccharomyces kudriavzevii* in wine fermentations. *FEMS Yeast Res.* 6 1221–1234. 10.1111/j.1567-1364.2006.00126.x 17156019

[B57] Grieco F. Carluccio M. A. Giovinazzo G. (2019). Autochthonous *Saccharomyces cerevisiae* starter cultures enhance polyphenols content, antioxidant activity, and anti-inflammatory response of Apulian red wines. *Foods* 8:453. 10.3390/foods8100453 31590278 PMC6836090

[B58] Herderich M. J. Siebert T. E. Parker M. Capone D. L. Mayr C. Zhang P. (2013). Synthesis of the ongoing works on rotundone, an aromatic compound responsible of the peppery notes in wines. *Internet J. Enol. Vitic.* 6 1–6.

[B59] Izquierdo-Cañas P. M. García-Romero E. Mena-Morales A. Gómez-Alonso S. (2016). Effects of malolactic fermentation on colour stability and phenolic composition of Petit Verdot red wines. *Wine Stud.* 5:5795. 10.4081/ws.2016.5795

[B60] Jaganath I. B. Crozier A. (2010). “Dietary flavonoids and phenolic compounds,” in *Plant Phenolics and Human Health*, ed. Fraga C. G. (Hoboken, NY: John Wiley & Sons, Inc).

[B61] Jagatić Korenika A. M. Tomaz I. Preiner D. Plichta V. Jeromel A. (2021). Impact of commercial yeasts on phenolic profile of Plavac Mali wines from Croatia. *Fermentation* 7:92. 10.3390/fermentation7020092

[B62] Jolly N. P. Varela C. Pretorius I. S. (2014). Not your ordinary yeast: non-Saccharomyces yeasts in wine production uncovered. *FEMS Yeast Res.* 14 215–237. 10.1111/1567-1364.12111 24164726

[B63] Klis F. M. Boorsma A. De Groot P. W. J. (2006). Cell wall construction in *Saccharomyces cerevisiae*. *Yeast* 23 185–202. 10.1002/yea.1349 16498706

[B64] Klis F. M. Mol P. Helligwerf K. Brul S. (2002). Dynamics of cell wall structure of *Saccharomyces cerevisiae*. *FEMS Microb. Rev.* 26 239–256. 10.1111/j.1574-6976.2002.tb00613.x 12165426

[B65] Kogani G. Pajtinka M. Babincova M. Miadokova E. Rauko P. Slamenova D. (2008). Yeast cell wall polysaccharides as antioxidants and antimutagens: can they fight cancer? Minireview. *Neoplasma* 55:387. 18665748

[B66] Kollár R. Reinhold B. B. Petráková E. Yeh H. J. Ashwell G. Drgonová J. (1997). Architecture of the yeast cell wall. Beta (1–>6)-glucan interconnects mannoprotein, beta(1–>)3-glucan, and chitin. *J Biol. Chem.* 272 17762–17775. 10.1074/jbc.272.28.17762 9211929

[B67] Legras J. L. Moreno-Garcia J. Zara S. Zara G. Garcia-Martinez T. Mauricio J. C. (2016). Flor Yeast: new perspectives beyond wine aging. *Front. Microbiol.* 7:503. 10.3389/fmicb.2016.00503 27148192 PMC4830823

[B68] Lencioni L. Taccari M. Ciani M. Domizio P. (2018). Zygotorulaspora florentina and *Starmerella bacillaris* in multistarter fermentation with *Saccharomyces cerevisiae* to reduce volatile acidity of high sugar musts. *Aust. J. Grape Wine Res.* 24 368–372. 10.1111/ajgw.12327

[B69] Lesage G. Bussey H. (2006). Cell wall assembly in Saccharomyces cerevisiae. *Microbiol. Mol. Biol. Rev.* 70 317–343. 10.1128/MMBR.00038-05 16760306 PMC1489534

[B70] Liu S. Laaksonen O. Yang B. (2019). Volatile composition of bilberry wines fermented with non-*Saccharomyces* and *Saccharomyces* yeasts in pure, sequential and simultaneous inoculations. *Food Microbiol.* 80 25–39. 10.1016/j.fm.2018.12.015 30704594

[B71] Liu S. Q. Pilone G. J. (2000). An overview of formation and roles of acetaldehyde in winemaking with emphasis on microbiological implications. *Intern. J. Food Sci. Technol.* 35 49–61. 10.1046/j.1365-2621.2000.00341.x

[B72] Lonvaud-Funel A. (1999). Lactic acid bacteria in the quality improvement and depreciation of wine. *Ant. Van Leeuw.* 76 317–331. 10.1023/A:1002088931106 10532386

[B73] Mansfield A. K. Zoecklein B. W. Whiton R. S. (2002). Quantification of glycosidase activity in selected strains of *Brettanomyces bruxellensis* and *Oenococcus oeni*. *Am. J. Enol. Vitic.* 53 303–307.

[B74] Manzanares P. Rojas V. Genovés S. Vallés S. (2000). A preliminar search for anthocyanin-ß-D-glucosidase activity in non-*Saccharomyces* wine yeasts. *Int. J. Food Sci. Technol.* 35 95–103. 10.1046/j.1365-2621.2000.00364.x

[B75] Mazauric J. P. Salmon J. M. (2005). Interactions between yeast lees and wine poly- phenols during simulation of wine aging: I. Analysis of remnant polyphenolic com- pounds in the resulting wines. *J. Agric. Food Chem.* 53 5647–5653. 10.1021/jf050308f 15998128

[B76] Mazauric J. P. Salmon J. M. (2006). Interactions between yeast lees and wine poly- phenols during simulation of wine aging: II. Analysis of desorbed polyphenol com- pounds from yeast lees. *J. Agric. Food Chem.* 54 3876–3881. 10.1021/jf060037o 16719509

[B77] Medina K. Boido E. Dellacassa E. Carrau F. (2005). Yeast interactions with anthocyanins during red wine fermentation. *Am. J. Enol. Vitic.* 56 104–109.

[B78] Mekoue Nguela J. Vernhet A. Julien-Ortiz A. Sieczkowski N. Mouret J. R. (2019). Effect of grape must polyphenols on yeast metabolism during alcoholic fermentation. *Food Res. Int.* 121 161–175. 10.1016/j.foodres.2019.03.038 31108737

[B79] Merida J. Lopez-Toledano A. Marquez T. Millan C. Ortega J. M. Medina M. (2005). Retention of browning compounds by yeasts involved in the winemaking of sherry type wines. *Biotechnol. Lett.* 27 1565–1570. 10.1007/s10529-005-1795-9 16245175

[B80] Morata A. Benito S. Loira L. Palomero E González M. C. Suárez-Lepe J. A. (2012). Formation of pyranoanthocyanins by *Schizosaccharomyces pombe* during the fermentation of red must. *Int. J. Food Microbiol.* 159, 47–53. 10.1016/j.ijfoodmicro.2012.08.007 22921967

[B81] Morata A. Gómez-Cordovés M. C. Calderón F. Suárez J. A. (2006). Effects of pH, temperature and SO_2_ on the formation of pyranoanthocyanins during red wine fermentation with two species of *Saccharomyces*. *Int. J. Food Microbiol.* 106 123–129. 10.1016/j.ijfoodmicro.2005.05.019 16225947

[B82] Morata A. Gómez-Cordovés M. C. Colomo B. Suárez J. A. (2003). Pyruvic acid and acetaldehyde production by different strains of *Saccharomyces cerevisiae*: relationship with Vitisin A and B formation in red wines. *J. Agric. Food Chem.* 51 7402–7409. 10.1021/jf0304167 14640591

[B83] Morata A. González C. Suárez-Lepe J. A. (2007). Formation of vinylphenolic pyranoanthocyanins by selected yeasts fermenting red grape musts supplemented with hydroxycinnamic acids. *Int. J. Food Microbiol.* 116 144–152. 10.1016/j.ijfoodmicro.2006.12.032 17303275

[B84] Morata A. Loira I. Escott C. del Fresno J. M. Bañuelos M. A. Suárez-Lepe J. A. (2019). Applications of Metschnikowia pulcherrima in wine biotechnology. *Fermentation* 5:63. 10.3390/fermentation5030063

[B85] Morata A. Loira I. Heras J. M. Callejo M. J. Tesfaye W. González C. (2016). Yeast influence on the formation of stable pigments in red winemaking. *Food Chem.* 197 686–691. 10.1016/j.foodchem.2015.11.026 26617004

[B86] Nardi T. (2020). Microbial resources as a tool for enhancing sustainability in winemaking. *Microorganisms* 8:507. 10.3390/microorganisms8040507 32252445 PMC7232173

[B87] Nguyen T. H. Fleet G. H. Rogers P. L. (1998). Composition of the cell walls of several yeast species. *Appl. Microbiol. Biotechnol.* 50 206–212. 10.1007/s002530051278 9763691

[B88] Olejar K. J. Fedrizzi B. Kilmartin P. A. (2015). Influence of harvesting technique and maceration process on aroma and phenolic attributes of Sauvignon blanc wine. *Food Chem.* 183 181–189. 10.1016/j.foodchem.2015.03.040 25863627

[B89] Padilla B. Gil J. V. Manzanares P. (2016). Past and future of Non-*Saccharomyces* yeasts: from spoilage microorganisms to biotechnological tools for improving wine aroma complexity. *Front. Microbiol.* 7:411. 10.3389/fmicb.2016.00411 27065975 PMC4814449

[B90] Palomero F. Morata A. Benito S. Calderón F. Suárez-Lepe J. A. (2009). New genera of yeasts for over-lees aging of red wine. *Food Chem.* 112 432–441. 10.1016/j.foodchem.2008.05.098

[B91] Peter J. De Chiara M. Friedrich A. Yue J. X. Pflieger D. Bergström A. (2018). Genome evolution across 1,011 *Saccharomyces cerevisiae* isolates. *Nature* 556 339–344. 10.1038/s41586-018-0030-5 29643504 PMC6784862

[B92] Petruzzi L. Baiano A. De Gianni A. Sinigaglia M. Corbo M. R. Bevilacqua A. (2015). Differencial adsorption of Ochratoxin A and anthocyanins by inactivated yeasts and yeast cell walls during simulation of wine aging. *Toxins* 7 4350–4365. 10.3390/toxins7104350 26516913 PMC4626738

[B93] Pfliegler W. P. Pusztahelyi T. Pocsi I. (2015). Mycotoxins - prevention and decontamination by yeasts. *J. Basic Microbiol.* 55 805–818. 10.1002/jobm.201400833 25682759

[B94] Renault P. Coulon J. Moine V. Thibon C. Bely M. (2016). Enhanced 3-sulfanylhexan-1-ol production in sequential mixed fermentation with *Torulaspora delbrueckii*/*Saccharomyces cerevisiae* reveals a situation of synergistic interaction between two industrial strains. *Front. Microbiol.* 7:293. 10.3389/fmicb.2016.00293 27014216 PMC4792154

[B95] Renault P. Miot-Sertier C. Marullo P. Lagarrigue L. Lonvaud-Funel A. Bely M. (2009). Genetic characterization and phenotypic variability in *Torulaspora delbrueckii* species: potential applications in the wine industry. *Int. J. Food Microbiol.* 134 201–210. 10.1016/j.ijfoodmicro.2009.06.008 19619911

[B96] Ribereau-Gayon P. Glories Y. Maujean A. (2006). *Handbook of Enology.* West Sussex: John Wiley & Sons, Ltd. 10.1002/0470010398

[B97] Rinaldi A. Coppola M. Moio L. (2019). Aging of aglianico and sangiovese wine on mannoproteins: effect on astringency and color. *LWT Food Sci. Technol.* 105 233–241. 10.1016/j.lwt.2019.02.034

[B98] Romano P. Suzzi G. (1993). “Sulphur dioxide and wine microorganisms,” in *Wine Microbiology and Biotechnology*, ed. Fleet Harwood G. (Chur: Academic Publisher GmbH).

[B99] Sadoudi M. Tourdot-Maréchal R. Rousseaux S. Steyer D. Gallardo-Chacón J. J. Ballester J. (2012). Yeast–yeast interactions revealed by aromatic profile analysis of Sauvignon Blanc wine fermented by single or co-culture of non-*Saccharomyces* and *Saccharomyces yeasts*. *Food Microbiol.* 32 243–253. 10.1016/j.fm.2012.06.006 22986187

[B100] Samoticha J. Wojdyło A. Chmielewska J. Nofer J. (2019). Effect of different yeast strains and temperature of fermentation on basic enological parameters, polyphenols and volatile compounds of Aurore white wine. *Foods* 8:599. 10.3390/foods8120599 31757009 PMC6963419

[B101] Schiavone M. Déjean S. Sieczkowski N. Castex M. Dague E. François J. M. (2017). Integration of biochemical, biophysical and transcriptomics data for investigating the structural and nanomechanical properties of the yeast cell wall. *Front. Microbiol.* 8:1806. 10.3389/fmicb.2017.01806 29085340 PMC5649194

[B102] Schiavone M. Vax A. Formosa C. Martin-Yken H. Dague E. Francois J. M. (2014). A combined chemical and enzymatic method to determine quantitatively the polysaccharide components in the cell wall of yeasts. *FEMS Yeast Res.* 14 933–947. 10.1111/1567-1364.12182 25041403

[B103] Schwarz M. Wabnitz T. C. Winterhalter P. (2003). Pathway leading to the formation of anthocyanin-vinylphenol adducts and related pigments in red wines. *J. Agric. Food Chem.* 51 3682–3687. 10.1021/jf0340963 12769545

[B104] Setati M. E. Jacobson D. Andong U. C. Bauer F. F. (2012). The vineyard yeast microbiome, a mixed model microbial map. *PLoS One* 7:e52609. 10.1371/journal.pone.0052609 23300721 PMC3530458

[B105] Simonin S. Alexandre H. Nikolantonaki M. Coelho C. Tourdot-Maréchal R. (2018). Inoculation of *Torulaspora delbrueckii* as a bio-protection agent in winemaking. *Food Res. Int.* 107 451–461. 10.1016/j.foodres.2018.02.034 29580506

[B106] Stanley D. Bandara A. Fraser S. Cambers P. J. Stanley G. A. (2010). The ethanol stress response and ethanol tolerance of *Saccharomyces cerevisiae*. *J. Appl. Microbiol.* 109 13–24. 10.1111/j.1365-2672.2009.04657.x 20070446

[B107] Sumby K. Bartle L. Grbin P. Jiranek V. (2019). Measures to improve wine malolactic fermentation. *Appl. Microbiol. Biotechnol.* 103 2033–2051. 10.1007/s00253-018-09608-8 30648191

[B108] Suzzi G. Romano P. Zambonelli C. (1985). Saccharomyces strain selection in minimizing SO2 requirement during vinification. *Am. J. Enol. Vitic.* 36 199–202.

[B109] Timberlake C. F. Bridle P. (1976). Interactions between anthocyanins, phenolic compounds, and acetaldehyde and their significance in red wines. *Am. J. Enol. Vitic.* 27 97–105.

[B110] Tofalo R. Patrignani F. Lanciotti R. Perpetuini G. Schirone M. Di Gianvito P. (2016). Aroma profile of Montepulciano d’Abruzzo wine fermented by single and co-culture starters of autochthonous *Saccharomyces* and non-*Saccharomyces* yeasts. *Front. Microbiol.* 7:610. 10.3389/fmicb.2016.00610 27199939 PMC4848713

[B111] Tofalo R. Schirone M. Torriani S. Rantsiou K. Cocolin L. Perpetuini G. (2012). Diversity of *Candida zemplinina* strains from grapes and Italian wines. *Food Microbiol.* 29 18–26. 10.1016/j.fm.2011.08.014 22029914

[B112] Van Breda V. Jolly N. Van Wyk J. (2013). Characterisation of commercial and natural *Torulaspora delbrueckii* wine yeast strains. *Int. J. Food Microbiol.* 163 80–88. 10.1016/j.ijfoodmicro.2013.02.011 23558190

[B113] Vasserot Y. Caillet S. Maujean A. (1997). Study of anthocyanin adsorption by yeast lees. Effect of some physicochemical parameters. *Am. J. Enol. Vitic.* 48 433–437.

[B114] Vejarano R. (2020). Non-*Saccharomyces* in winemaking: source of mannoproteins, nitrogen, enzymes, and antimicrobial compounds. *Fermentation* 6:76. 10.3390/fermentation6030076

[B115] Verstrepen K. J. Fink G. R. (2009). Genetic and epigenetic mechanisms underlying cell-surface variability in protozoa and fungi. *Annu. Rev. Genet.* 43:1. 10.1146/annurev-genet-102108-134156 19640229

[B116] Vilela A. (2018). *Lachancea thermotolerans*, the non-*Saccharomyces* yeast that reduces the volatile acidity of wines. *Fermentation* 4:56. 10.3390/fermentation4030056

[B117] Virdis C. Sumby K. Bartowsky E. Jiranek V. (2021). Lactic acid bacteria in wine: technological advances and evaluation of their functional role. *Front. Microbiol.* 11:612118. 10.3389/fmicb.2020.612118 33519768 PMC7843464

[B118] Visioli F. Panaite S. A. Tomé-Carneiro J. (2020). Wine’s phenolic compounds and health: a pythagorean view. *Molecules* 25:4105. 10.3390/molecules25184105 32911765 PMC7570485

[B119] Wang S. Li S. Zhao H. Gu P. Chen Y. Zhang B. (2018). Acetaldehyde released by *Lactobacillus plantarum* enhances accumulation of pyranoanthocyanins in wine during malolactic fermentation. *Food Res. Int.* 108 254–263. 10.1016/j.foodres.2018.03.032 29735055

[B120] Waterhouse A. L. Sacks G. L. Jeffery D. W. (2016). *Understanding Wine Chemistry.* New York, NY: JohnWiley & Sons, Incorporated. 10.1002/9781118730720

[B121] Yin Q. Y. De Groot P. W. J. De Jong L. Klis F. M. De Koster C. G. (2007). Mass spectrometric quantitation of covalently bound cell wall proteins in *Saccharomyces cerevisiae*. *FEMS Yeast Res.* 7 887–896. 10.1111/j.1567-1364.2007.00272.x 17617218 PMC2040195

[B122] Yue X. F. Jing S. S. Ni X. F. Zhang K. K. Fang Y. L. Zhang Z. W. (2021). Anthocyanin and phenolic acids contents influence the color stability and antioxidant capacity of wine treated with mannoprotein. *Front. Nutr.* 8:691784. 10.3389/fnut.2021.691784 34222310 PMC8249586

[B123] Zara S. Gross M. K. Zara G. Budroni M. Bakalinsky A. T. (2010). Ethanol-independent biofilm formation by a flor wine yeast strain of *Saccharomyces cerevisiae*. *Appl. Environ. Microbiol.* 76 4089–4091. 10.1128/AEM.00111-10 20435772 PMC2893507

[B124] Zhang P. Ma W. Meng Y. Zhang Y. Jin G. Fang Z. (2021). Wine phenolic profile altered by yeast: mechanisms and influences. *Compr. Rev. Food Sci. Food Saf.* 20 3579–3619. 10.1111/1541-4337.12788 34146455

